# Network pharmacological analysis and *in vitro* testing of the rutin effects on triple-negative breast cancer

**DOI:** 10.1515/med-2024-1079

**Published:** 2025-01-08

**Authors:** Cheng Chang, Ruiying Jia, Bin Fang, Yaoyao Miao, Lili Zhang

**Affiliations:** General Surgery Department, Qingdao Hospital, University of Health and Rehabilitation Sciences (Qingdao Municipal Hospital), Qingdao, 266011, China; Surgical Center, Qingdao Hospital, University of Health and Rehabilitation Sciences (Qingdao Municipal Hospital), Qingdao, 266011, China; Colorectal Surgery Department, Affiliated Qingdao Hiser Hospital of Qingdao University (Qingdao Hospital of Traditional Chinese Medicine), Qingdao, 266011, China; Pulmonary Disease Department, Affiliated Qingdao Hiser Hospital of Qingdao University (Qingdao Hospital of Traditional Chinese Medicine), Qingdao, 266011, China; Imaging Department, Affiliated Qingdao Hiser Hospital of Qingdao University (Qingdao Hospital of Traditional Chinese Medicine), Qingdao, 266011, China

**Keywords:** rutin, triple-negative breast cancer, network pharmacology, mechanism

## Abstract

**Objectives:**

This study aims to assess the potential mechanism of rutin to treat triple-negative breast cancer (TNBC) based on network pharmacology followed by *in vitro* experiments.

**Methods:**

The potential rutin targets were predicted, and the DisGeNET database was used to obtain the disease targets. The intersection targets were identified with Venny 2.1 software, with the String database subsequently used as input to produce the “drug-target-disease” visual network employing Cytoscape 3.7.2. Gene ontology. Kyoto Encyclopaedia of Genes and Genomes analyses were performed for intersection targets, while AutoDock Vina was used for molecular docking and visualization. Cell viability was assessed using the Colorimetric CCK-8 test, and apoptosis was analyzed using PI/Annexin V. The predicted core targets were confirmed by qPCR and western blotting assays.

**Results:**

EGFR, IL6, TNF, and INS were found as the primary targets. The molecular docking analysis revealed the rutin interaction with the core targets. The *in vitro* results confirmed that rutin inhibited the growth of the MDA-MB-231 cell line. Rutin also induced cell death and decreased the expressions of IL6, TNF, INS, and EGFR.

**Conclusion:**

Rutin’s multi-target effects and molecular mechanism for treating TNBC were confirmed through preliminary results. The results provide a theoretical base for rutin’s possible function in breast cancer treatment.

## 1 Introduction

Breast cancer is the most common malignant tumor of females in the world [1]. Triple-negative breast cancer (TNBC) covers about 15–20% of the disease incidence among its various subtypes. Because TNBC lacks hormone receptors such as HER2, PR, and estrogen receptors, as well as progesterone and estrogen [2], it is the most challenging subtype of breast cancer to treat [3]. This leads to a poor prognosis, high rates of recurrence and metastasis, and a high mortality rate. Consequently, breast cancer research has attracted the attention of researchers in recent years.

In particular, there has been significant interest in utilizing plant-derived natural products as anti-cancer medications due to their ability to target various sites and the high effectiveness and minimal toxicity of their active components. Rutin, a flavonoid component, is often present in fruits and vegetables [4], with buckwheat being the primary source [5]. The name rutin is derived from the *Ruta graveolens* L. plant. Its physical characteristics present it as yellowish-green needle-shaped crystals. It is well known to possess several pharmacological properties, potentially preventing neuroinflammation and high cholesterol levels.

Along with other biological activities, it also serves as an anti-plasma, anti-arthritis, anti-viral, anti-hypertension, anti-injury, anti-bacterial, anti-inflammatory, and anti-cancer agent [6,7,8,9,10,11,12]. In recent years, the anti-tumor properties of rutin have become a research hotspot [13,14,15,16]. Studies have also shown the substantial efficacy of rutin in the treatment of TNBC. However, further research is required to investigate and clarify its mode of action.

**Figure 1:**
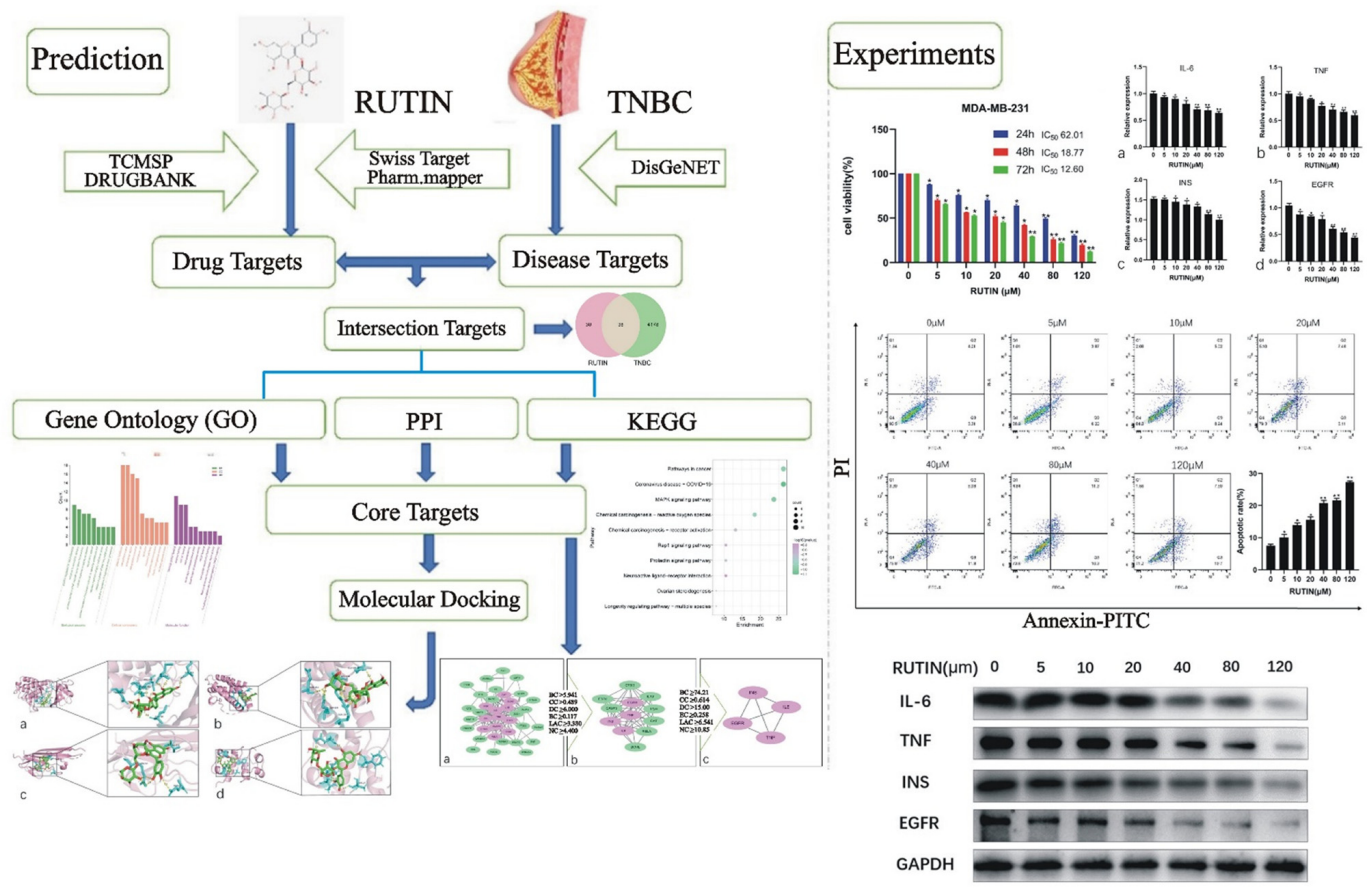
The study workflow is based on network pharmacology, aiming to determine rutin’s possible pharmacological action in treating TNBC.

Network pharmacology, a field rooted in systems biology, examines the correlation between “drug–target–disease” and highlights the stimulation of many regulatory pathways [17]. This approach introduces a novel framework for investigating intricate connections across diseases, diverse targets, and drugs. The present study employed the network pharmacological technique to anticipate the potential targets of rutin. The cell-based experimentation also verified the possible targets to explain its mechanism of action in treating TNBC (Figure 1).

## 2 Methodology

### 2.1 Drug and disease target screening

The TCMSP and Swiss Target Prediction tools and the DRUGBANK and PharmMapper databases were used to predict rutin’s possible targets. The UniProt database standardized the target information. A search was conducted in the DisGeNET database using the keyword “Triple Negative Breast Cancer.” The score value indicated the degree of correlation between the target and the disease. Targets with scores higher than the median were selected as the disease targets for breast cancer. The ultimate disease target was obtained by removing the duplicates.

### 2.2 Intersection target acquisition and action network construction

The obtained drug and disease targets were used to identify the common targets by importing these to Venny 2.1 software. A Venn diagram was drawn for the intersecting targets of rutin in TNBC. These selected targets were then imported into Cytoscape 3.7.2 for visualization before constructing the drug–target–disease action network diagram.

### 2.3 Kyoto Encyclopaedia of Genes and Genomes (KEGG) pathway and gene function analysis

Gene ontology (GO) enrichment analysis was conducted for the intersecting targets of the drug and the disease to elucidate the biological functions of rutin on TNBC targets. All GO terms were assessed, i.e., biological processes, molecular functions, and cell components. Moreover, the typical targets were also mapped to the DAVID database for KEGG enrichment analysis to identify the signal pathways related to drug action.

### 2.4 Protein–protein interaction (PPI) network establishment and analysis

The interaction network diagram was acquired to analyze the protein–protein interaction via the String database. The TSV data were subsequently extracted from the PPI before their analysis with Cytoscape 3.7.2 and CytoNCA. Network centralities (NC), local average connectivity-based method (LAC), eigenvector centralities (EC), degree centralities (DC), closeness centralities (CC), and betweenness centralities (BC) were carried out on the network topology as well as on the key target genes while filtering to obtain the core target genes. The filtering criteria were adjusted according to the median values of NC, LAC, EC, DC, CC, and BC.

### 2.5 Molecular docking

The relationship between the core targets and rutin was verified by selecting the core targets in the protein–protein interaction network for molecular docking. Initially, the PDB database was utilized to acquire the structure of the central target proteins, subsequently eliminating water and small molecules. The protein was hydrogenated, and its charge was calculated using AutoDock Tools (version 1.5.6). The Rutin structure was then taken from the PubChem database and analyzed using AutoDock Tools to observe charge balance and rotatable bonds. Finally, AutoDock Vina was used to determine the receptor–ligand docking. The structure with the highest binding affinity, i.e., corresponding to the least free binding energy, was selected before visualization with PyMol (version 2.4.1) software.

### 2.6 *In vitro* experimentation

#### 2.6.1 Cell viability analysis

The CCK-8 kit assay was used to examine the effects of rutin (product number, drk-0447; purity ≥ 92.5%; molecular weight is 610.51; Shanxi Sayer Biological Co. Ltd., China) on the TNBC cell line MDA-MB-231 (Cell Resources Center, Shanghai Academy of Biological Sciences, Chinese Academy of Sciences). The cells were cultured in 96-well plates with a density of 8 × 10^3^ cells/100 µL before being treated with various concentrations (0, 5, 10, 20, 40, 80, and 120 μM) of rutin for 24, 48, and 72 h. A 10 µL solution of CCK-8 was added to the wells and incubated for 90 min. The plates were optically scanned at a wavelength of 450 nm using a microplate reader. The concentration at which 50% inhibition occurred (IC_50_) was calculated.

#### 2.6.2 Apoptosis assay

MDA-MB-231 cell lines at the log growth phase were digested and washed. The cells were disseminated at a medium density (1 × 10^5^ cells/mL). Moreover, 2 mL of the cell suspension was added to each well of culture plates and incubated overnight to facilitate cell adherence. Different concentrations of rutin (0, 5, 10, 20, 40, 80, and 120 μM) were added, and plates were incubated further for 2 days. Next, the cells were digested, washed, and subjected to apoptosis assay as per the instructions of the apoptosis kit. Briefly, 500 μL of the buffer was used to make cell suspension, followed by incubation with PI (5 μL) and Annexin V-FITC (5 μL) for the next 15 min in the dark. The samples were detected for apoptosis via a flow cytometer.

#### 2.6.3 mRNA and protein detection of core targets

Gene expression analysis was performed by RT-qPCR. Cells were grown in six-well culture plates while maintaining a seeding density of 2 × 10^5^ cells/well. Total RNA extraction was performed employing the Trizol approach. The RNA purity and concentration were then quantified using a UV spectrophotometer before the reverse transcription of *IL6, TNF, INS*, and *EGFR* mRNA. qPCR was performed using the resultant cDNA as a template for amplification. The overall reaction volume was set to 20 µL, consisting of 10 µL of Master Mix (SYBR Green), 1 µL each of forward and reverse primers, 2 µL of cDNA, and 2 µL of NFW. The PCR conditions used were as follows:

Initial denaturation: 95°C for 10 min of 40 cycles

Denaturation: 95°C for 15 s

Annealing: 60°C for 30 s.

Amplification curves and Ct values were recorded, and GAPDH was used as the internal control. The equation to determine the relative gene expression level was 2^−ΔΔCT^. Primer sequences are provided in Table 3, and the results are shown in Figure 10.

#### 2.6.4 Western blotting

Each group’s cells were lysed for half an hour using PMSF-containing lysate. The suspension was spun at 12,000 rpm for 5 min at 4°C. The protein level in the supernatant was determined quantitatively using a BCA protein detection kit. The cells were treated with primary antibodies (anti-INS, EGFR, TNF, and IL6) and incubated overnight (4°C). The cells were gently washed and incubated with a secondary antibody (sheep anti-rabbit IgG) for 2 h at room temperature (RT). The image was acquired by chemiluminescence. GAPDH was employed as an internal reference/control.

### 2.7 Statistical analysis

The experimental data were statistically evaluated using Prism software (version 9.4). Data values are presented as mean ± SD. The group comparison was performed using the Student’s *t*-test, with *p*-values <0.05 considered statistically significant.

## 3 Result**s**

### 3.1 Drug and disease targets

Overall, 68 drug targets were identified after eliminating replicates: of these, 21 were from the TCMSP platform, 2 from the DRUGBANK database, 4 from the Swiss Target Prediction platform (probability = 1), and 41 from the PharmMapper database (norm fit value >0.9).

After searching on DisGeNET and filtering for scores higher than 0.5, 4,216 targets associated with TNBC were identified, taking into account the removal of duplicate entries.

### 3.2 Acquisition of intersecting targets and the construction of the action network

To identify potential targets of rutin in the context of breast cancer, we employed a systematic approach to intersect the targets associated with breast cancer and those predicted for rutin. Initially, we screened a comprehensive list of 4,216 breast cancer-related targets from the DisGeNET database. This robust resource aggregates data from multiple sources, including expert-curated databases, GWAS catalogs, and the scientific literature. This extensive list provided a broad spectrum of molecular targets implicated in the pathogenesis and progression of breast cancer.

**Figure 2:**
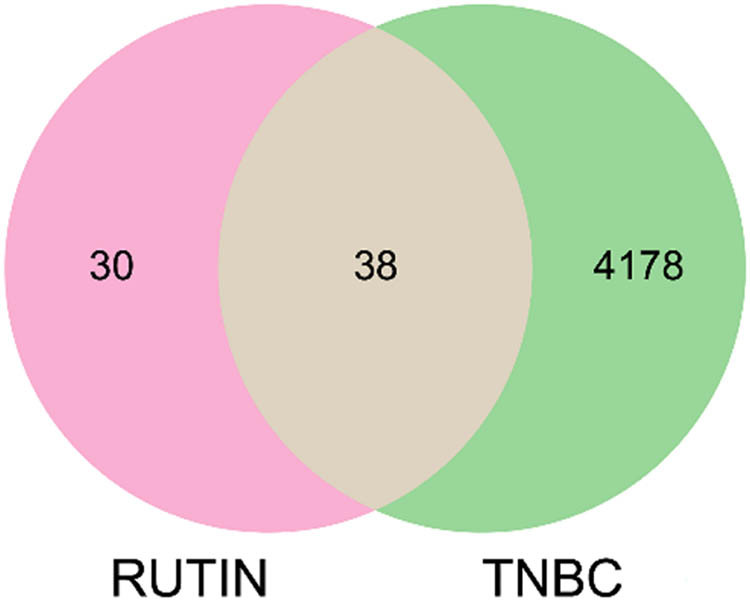
Venn diagram of the intersection of rutin and TNBC.

Simultaneously, we identified 68 potential targets of rutin through a combination of databases, including SwissTargetPrediction and STITCH, which predict drug–target interactions based on known chemical structures, ligand-based approaches, and existing experimental data. These targets represent the diverse biological activities of rutin, reflecting its potential to modulate various pathways involved in cancer biology (Figure 2).

The intersection of these two datasets revealed 38 common targets (Table 1), suggesting that these molecules may play a critical role in mediating the therapeutic effects of rutin in breast cancer. This intersection is crucial, as it narrows down the wide range of potential targets to those that are most relevant in the context of breast cancer, thus providing a focused framework for further investigation.

**Table 1:** Targets of rutin for the treatment of TNBC

Target name	Gene name	Uniprot ID
Transcription factor p65	RELA	Q04206
Tumor necrosis factor	TNF	P01375
Interleukin-6	IL6	P05231
Caspase-3	CASP3	P42574
NADPH–cytochrome P450 reductase	POR	P16435
Superoxide dismutase [Cu–Zn]	SOD1	P00441
Catalase	CAT	P04040
Interleukin-1 beta	IL1B	P01584
Protein kinase C beta type	PRKCB	P05771
Arachidonate 5-lipoxygenase	ALOX5	P09917
3-Hydroxy-3-methylglutaryl-coenzyme A reductase	HMGCR	P04035
Glutathione *S*-transferase P	GSTP1	P09211
Insulin	INS	P01308
Integrin beta-2	ITGB2	P05107
Thromboxane A2 receptor	TBXA2R	P21731
Aldo-keto reductase family 1 member C3	AKR1C3	P42330
Carbonyl reductase [NADPH] 1	CBR1	P16152
Alpha-2a adrenergic receptor	ADRA2A	P08913
Beta-secretase 1	BACE1	P56817
Butyrylcholinesterase	BCHE	P06276
Carbonic anhydrase 12	CA12	O43570
Carbonic anhydrase 2	CA2	P00918
Complement Factor B	CFB	P00751
Cathepsin D	CTSD	P07339
Epidermal growth factor receptor	EGFR	P00533
Estrogen receptor 2	ESR2	Q92731
Coagulation Factor II	F2	P00734
Glucosylceramidase beta	GBA	P04062
Mitogen-activated protein kinase 10	MAPK10	P53779
Matrix metallopeptidase 13	MMP13	P45452
Matrix metallopeptidase 3	MMP3	P08254
Phosphodiesterase 4B	PDE4B	Q07343
Progesterone neceptor	PGR	P06401
Purine nucleoside phosphorylase	PNP	P00491
Reticulon 4 receptor	RTN4R	Q9BZR6
Transforming growth factor beta receptor 2	TGFBR2	P37173
Thyroid hormone receptor beta	THRB	P10828
Transthyretin	TTR	P02766

**Figure 3:**
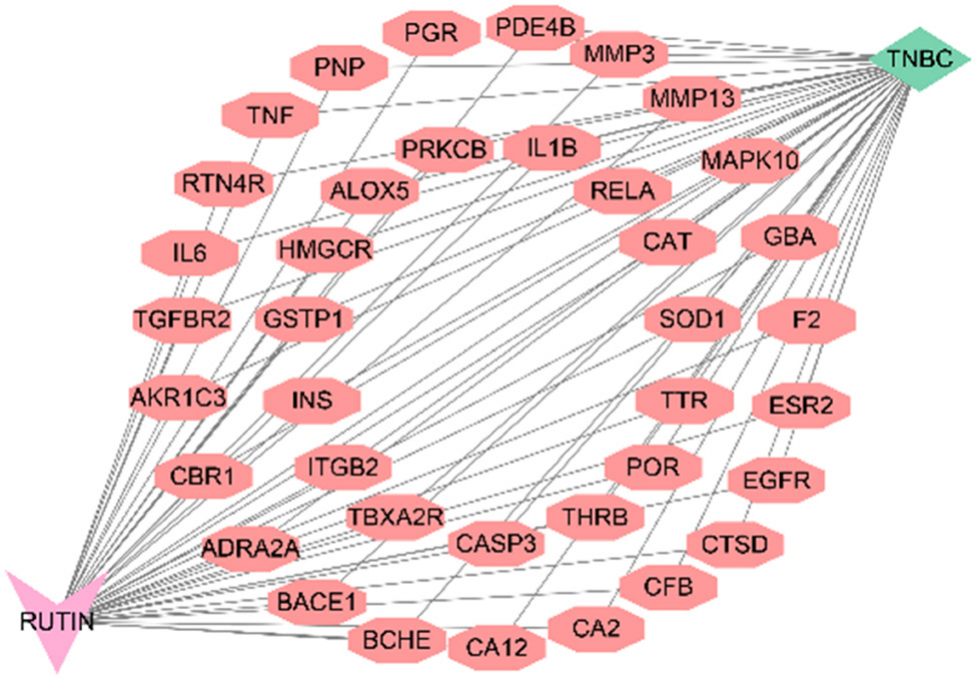
Drug–target–disease action network diagram of intersected targets.

We constructed a drug–target–disease action network diagram to visualize the complex relationships between rutin, its targets, and breast cancer. This network illustrates the interactions between rutin and the 38 intersecting targets, highlighting how these targets are linked to breast cancer. The nodes in the network represent the drug (rutin), the intersecting targets, and the disease (breast cancer), while the edges indicate the interactions between them (Figure 3). By mapping these interactions, the network provides a comprehensive overview of the potential mechanisms through which rutin may affect breast cancer. It offers valuable insights for future experimental validation and therapeutic development.

### 3.3 Enrichment analysis

**Figure 4:**
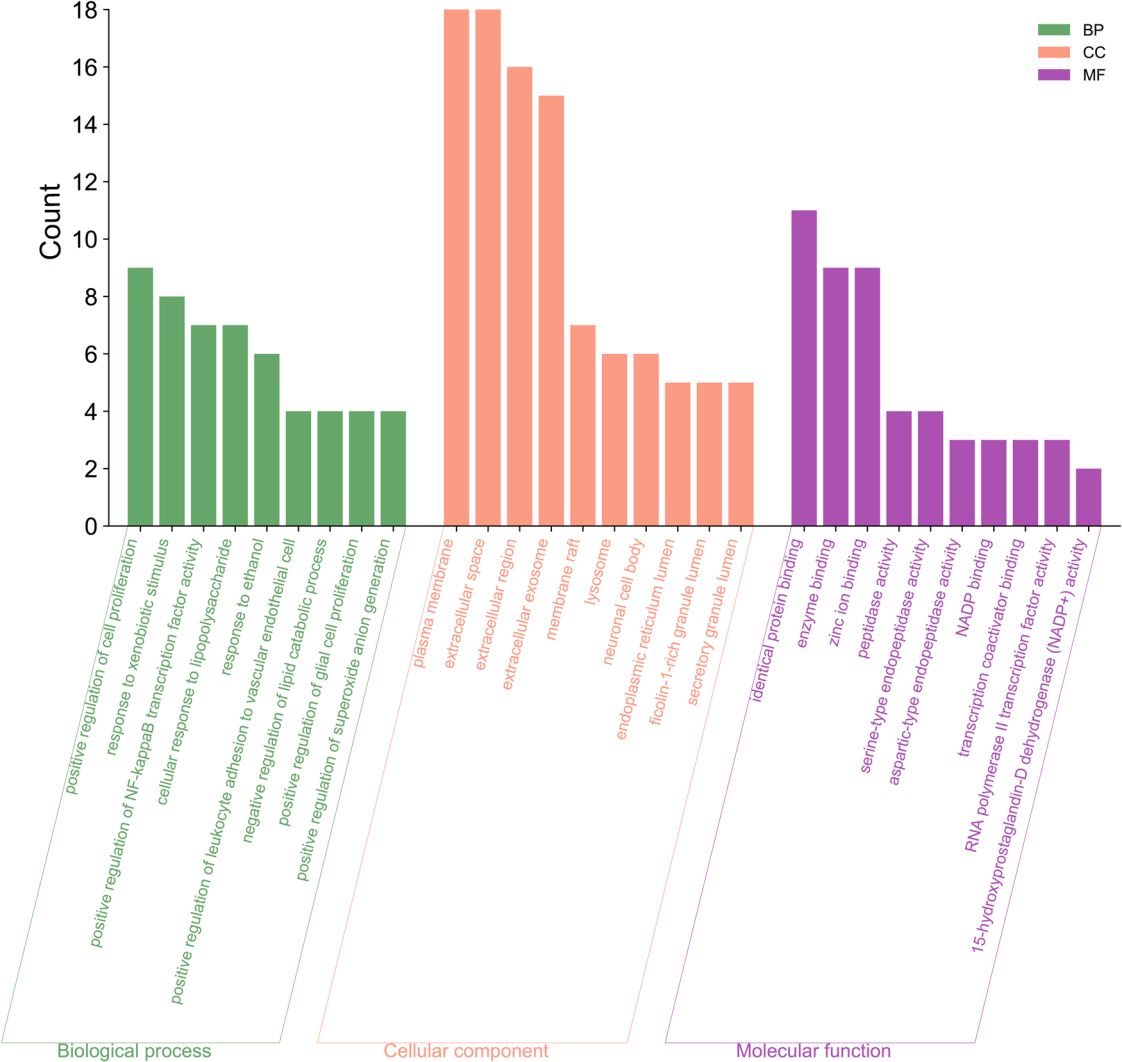
GO biological process analysis of intersected targets.

DAVID database was used to perform GO enrichment analysis on the typical targets of the drug and the disease. The results identified 220 GO terms, including 172 BP, 22 CC, and 26 MF. Based on the *p*-values, starting from the smallest one, the top 10 were selected, as shown in Figure 4. In terms of BP, these were primarily associated with drug response, positive regulation of cell division, and reaction to exogenous stimuli. The extracellular region, extracellular space, and plasma membrane were all simultaneously implicated in the enhanced cell components. Ultimately, the molecular roles of zinc ion binders, enzymes, and identical proteins were studied.

**Figure 5:**
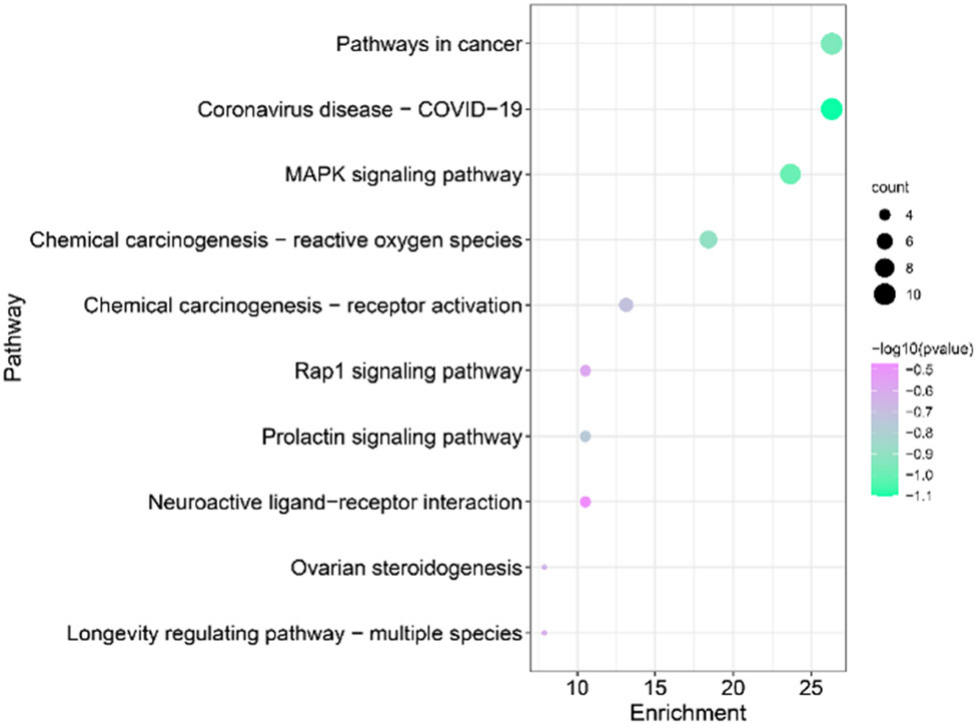
KEGG analysis of intersected targets.

The KEGG enrichment analysis was conducted by uploading these targets to the DAVID database to investigate the impact of rutin on TNBC. The top 10 were selected based on the *p*-values, starting from the lowest value, as depicted in Figure 5. The results presented a higher enrichment of genes in the tumor pathway (HSA05200) and MAPK signaling pathway (HSA04010).

### 3.4 PPI network establishment and analysis

**Figure 6:**
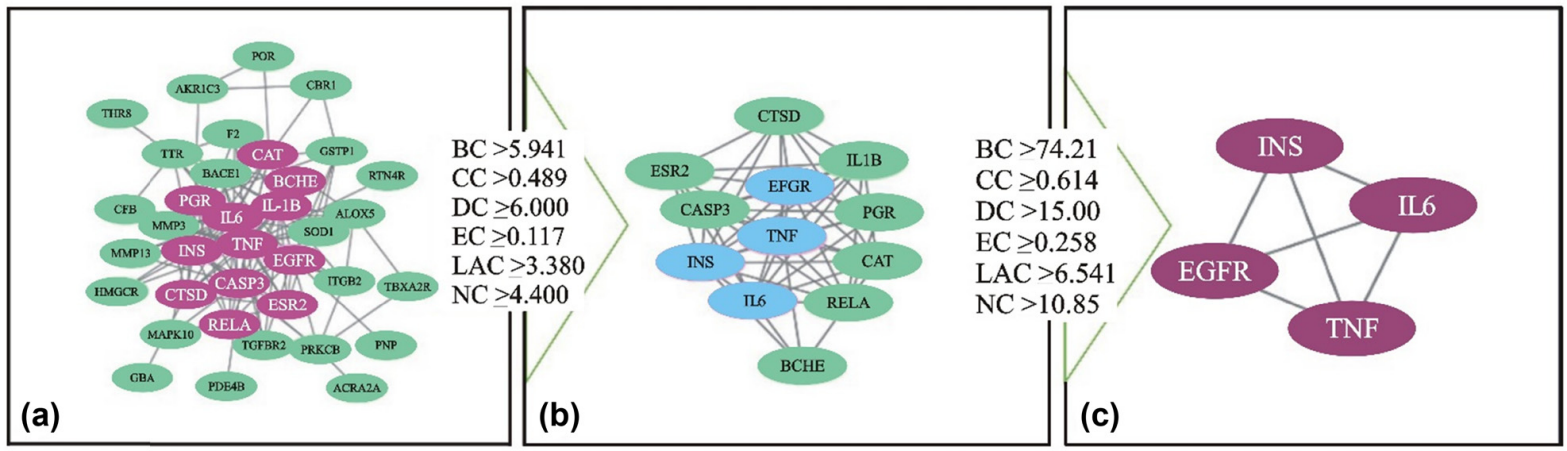
The procedure of topological screening for the PPI network. (a) 38 core targets, except CA2 and CA12, (b) 12 target genes, and (c) 4 target genes.

A total of 38 target proteins were uploaded to the STRING database for analysis. The species chosen for the investigation was “Homosapiens.” This resulted in generating a protein interaction network map consisting of 135 connections. CA2 and CA12 were among the target proteins not associated with interactions. The first topological conditions of the 38 core targets were BC ≥ 5.9416667, CC ≥ 0.48953442, DC ≥ 6, EC ≥ 0.117143418, LAC ≥ 3.3809524, and NC ≥ 4.4. Additionally, 12 target genes were obtained with 12 nodes and 51 edges. In the second topological analysis, the conditions were BC ≥ 74.212185, CC ≥ 0.614224, DC ≥ 15, EC ≥ 0.2583165, LAC ≥ 6.5416668, and NC ≥ 10.85518, with four target genes having four nodes and six edges also obtained. The findings are displayed in Figure 6.

### 3.5 Molecular docking

**Figure 7:**
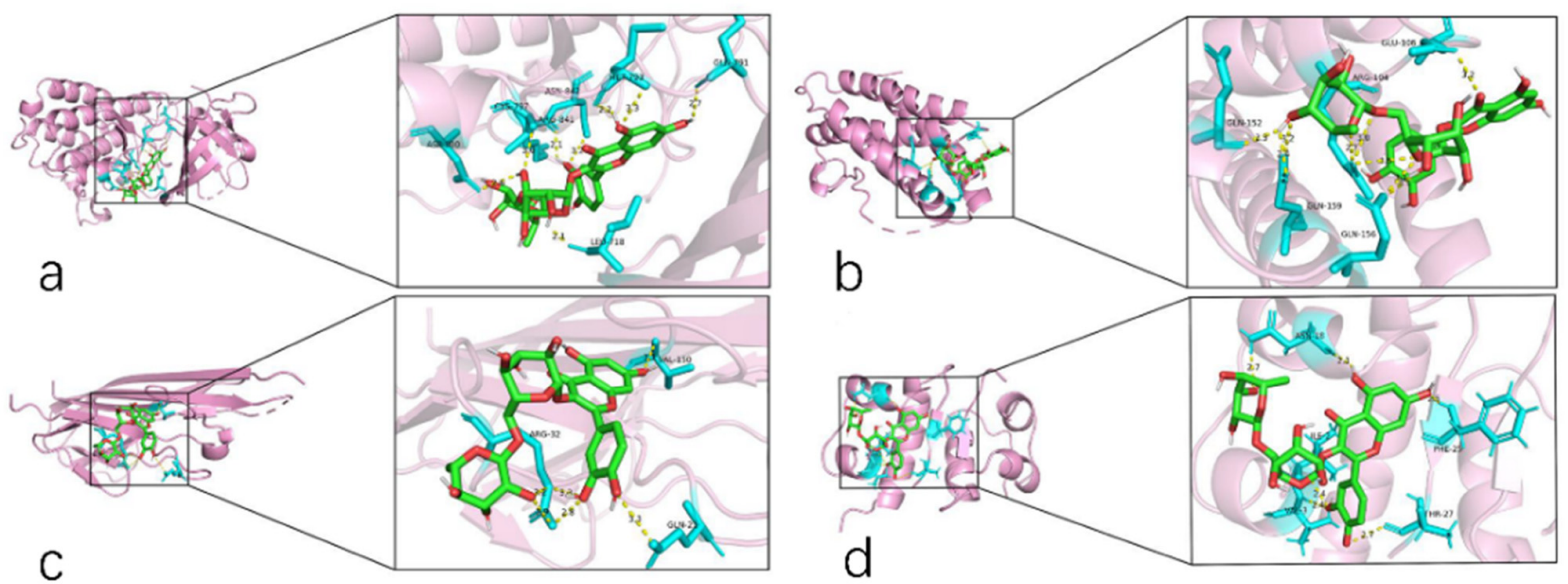
Rutin and core targets conformations: (a) EGFR, (b) IL6, (c) TNF, and (d) INS.

Molecular docking was used to analyze four core targets (IL6, TNF, INS, and EGFR) and rutin. It is generally believed that a docking score of <7 indicates that the target-7 has good binding activity with the active component, while a docking score of <5 indicates that the target-5 has good binding activity with the main protein target. The molecular docking results showed that the active compound’s conformation had excellent binding effects with the primary protein target and that the interaction was also dependable. Table 2 displays the binding affinity values, and Figure 7 shows the conformation of the main active drugs and targets.

**Table 2:** Molecular docking binding energy

PDB ID	Symbol	MOL_ID	Molecule_name	Energy (kcal/mol)
5UGC	EGFR	MOL000415	Rutin	−8.1
5UUI	TNF	MOL000415	Rutin	−7.0
1ALU	IL6	MOL000415	Rutin	−7.7
1BEN	INS	MOL000415	Rutin	−6.7

### 3.6 Molecular biological results

#### 3.6.1 Viability of MDA-MB-231 cells affected by rutin

**Figure 8:**
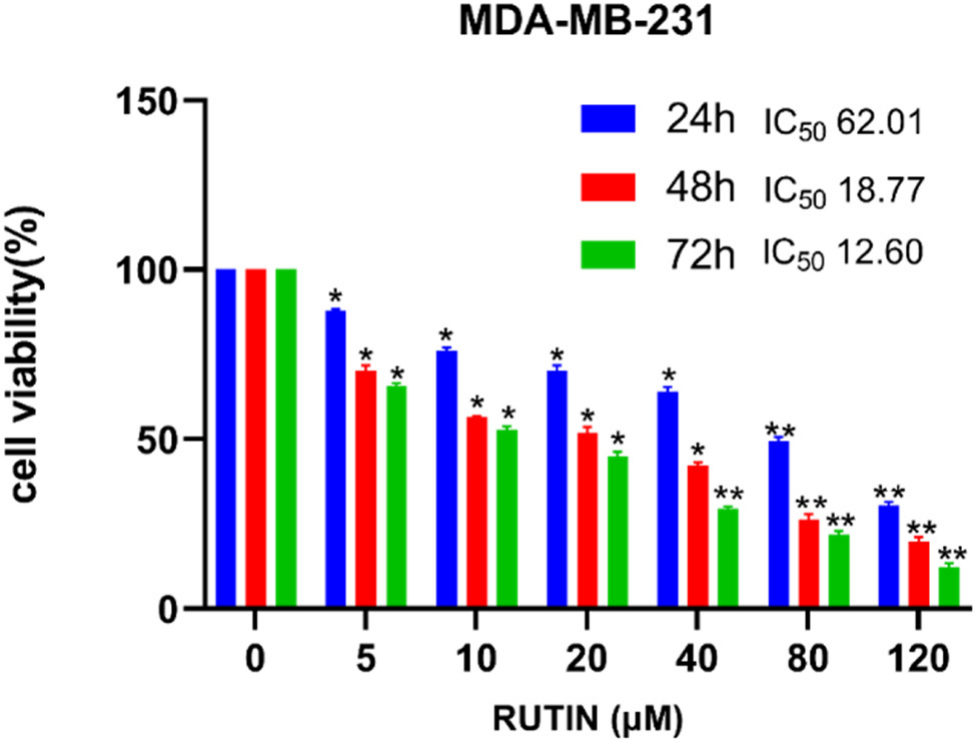
Impact of rutin on the viability of MDA-MB-231 cells. *P < 0.05 **P < 0.01 vs 0 Mm.

Rutin induced the proliferation of the TNBC cell line with increased time and concentration. The IC_50_ values of rutin at 24, 48, and 72 h were 12.60, 18.77 and 62.01, respectively. Furthermore, as presented in Figure 8, the IC_50_ value of rutin reduced significantly on the MDA-MB-231 cell line over time.

#### 3.6.2 Effect of rutin on the apoptosis

**Figure 9:**
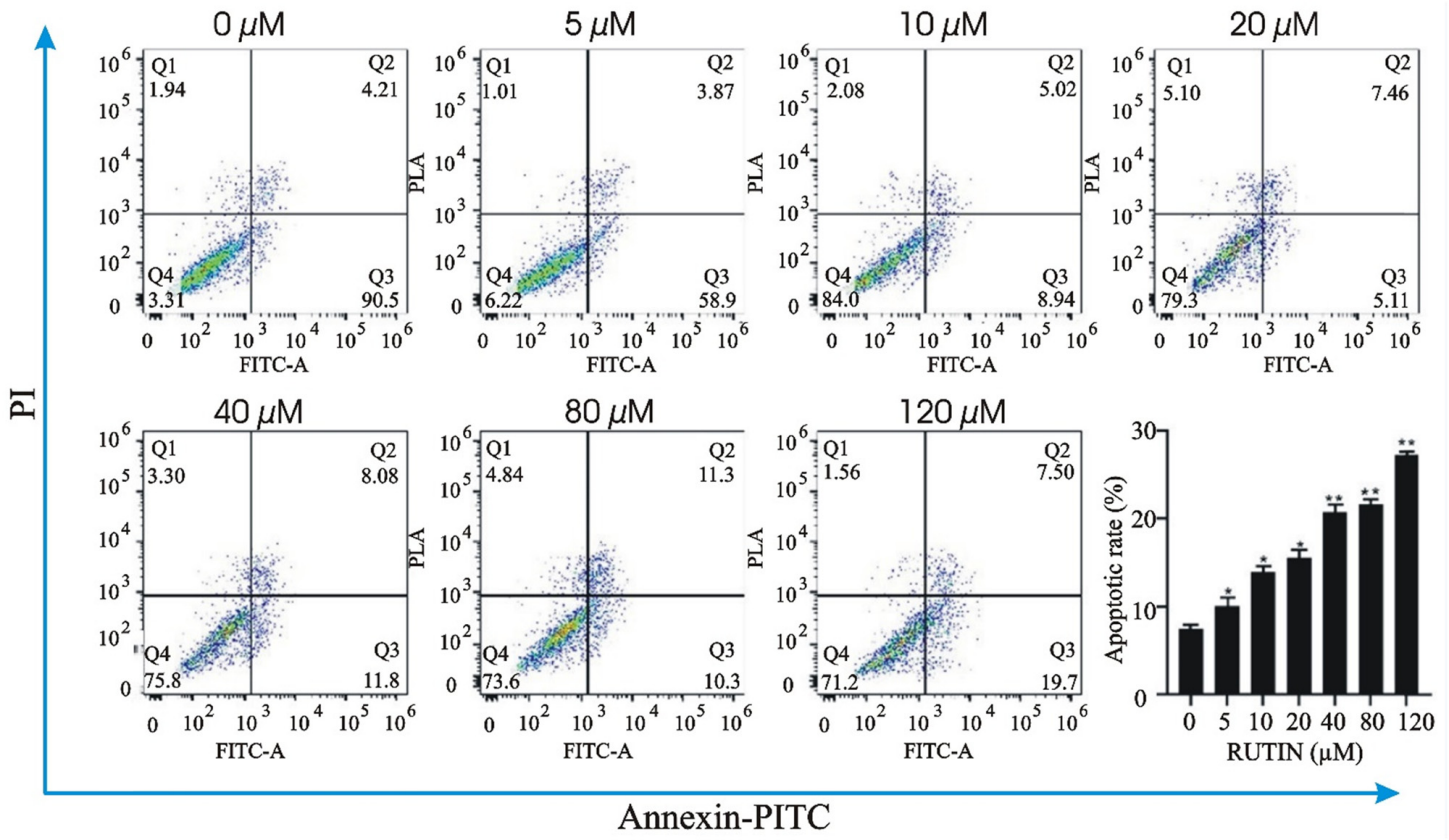
Effect of the different concentrations of rutin on the apoptosis of the MDA-MB-231 cell line.

Different concentrations of rutin were used to treat the MDA-MB-231 cell line. Results of PI/AnnexinV double staining and flow cytometry are shown in Figure 9. The cellular apoptosis was found to be increased from 10.09 to 27.29%, while that of the control 0 μM was only 7.52%. These results indicated that rutin could induce apoptosis in the MDA-MB-231 cell line.

#### 3.6.3 Regulation of the core target mRNA and protein by rutin

**Figure 10:**
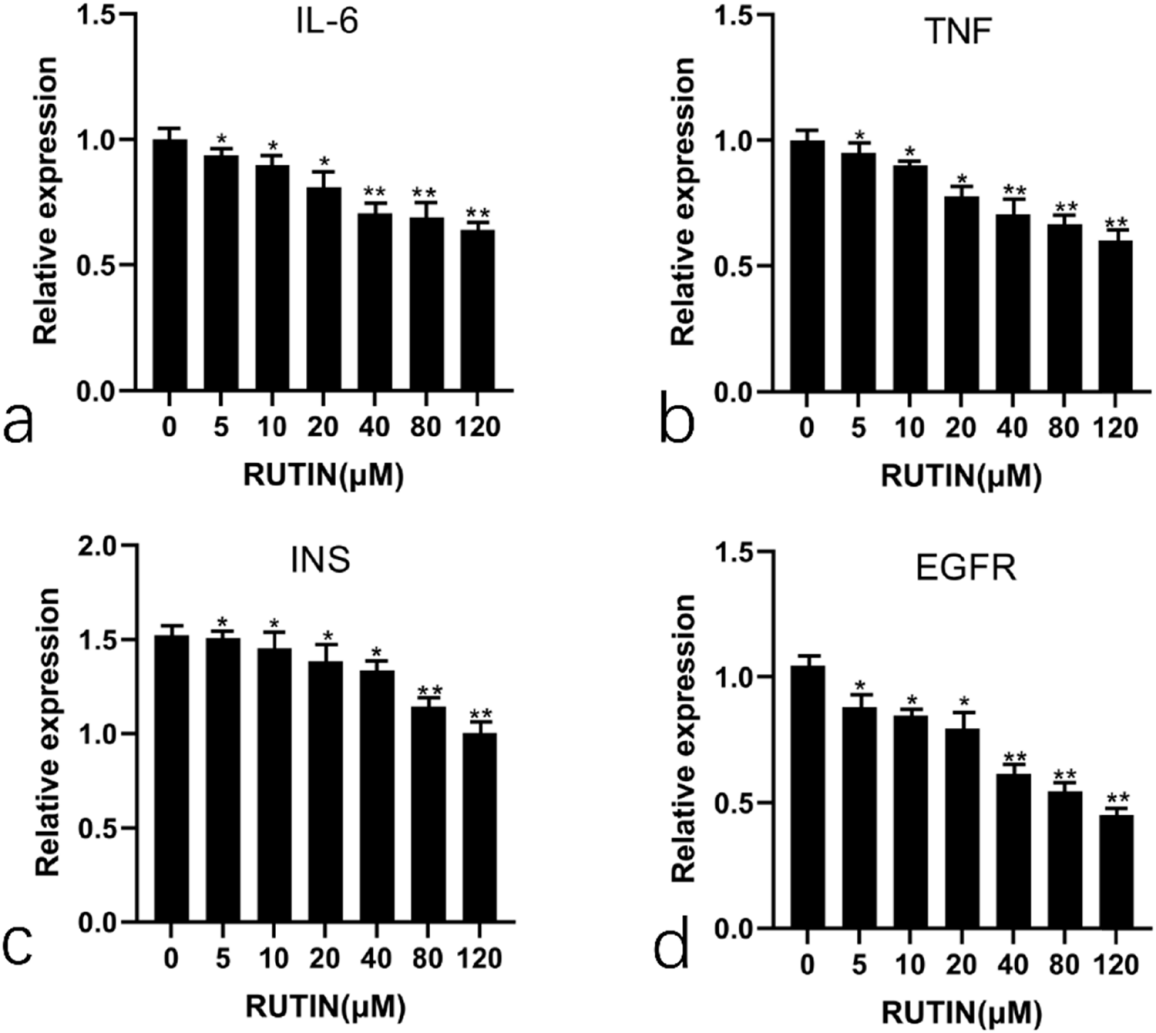
Genes expression analysis of (a) *IL6*, (b) *TNF*, (c) *INS*, and (d) *EGFR* in rutin-treated cells as examined by qPCR.

The mRNA expression levels of core targets *IL6, TNF, INS,* and *EGFR* were examined. The primer information is shown in Table 3. Rutin showed the reduced expression of these four genes. The results are shown in Figure 10.

**Table 3:** Primer information

Gene		Sequence (5′→3′)
IL-6	F	TGAGGAGACTTGCCTGGTGA
	R	CTGCACAGCTCTGGCTTGTT
TNF	F	CTGCACTTTGGAGTGATCGG
	R	TCAGCTTGAGGGTTTGCTAC
INS	F	TGAGGAGACTTGCCTGGTGA
	R	CTGCACAGCTCTGGCTTGTT
EGFR	F	GTCTGCCGCAAATTCCGAGA
	R	GACACTTCTTCACGCAGGT
GAPDH	F	GGAGCGAGATCCCTCCAAAAT
	R	GGCTGTTGTCATACTTCTCATGG

**Figure 11:**
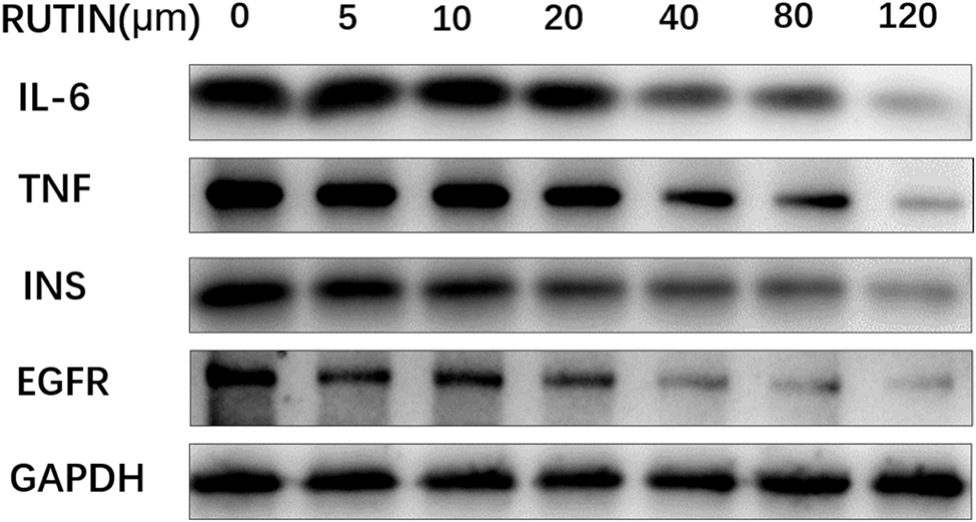
Protein expression levels of IL6, TNF, INS, EGFR, and GAPDH in cell cycle control in the MDA-MB-231 cell line treated with rutin.

Western blotting was conducted to analyze the protein expression of IL6, TNF, INS, and EGFR. The results demonstrated a significant decrease in the protein expression with increasing concentrations of rutin, as shown in Figure 11.

## 4 Discussion

While rutin has been widely studied, its role in cancer, particularly in TNBC, remains complex and not fully understood. Recent studies have explored the potential of flavonoid-like rutin in modulating various signaling pathways relevant to TNBC. For example, rutin has been implicated in inhibiting key oncogenic pathways, such as the PI3K/AKT/mTOR pathway, which is often dysregulated in TNBC [18,19,20]. Our network pharmacological analysis has identified several targets, including PIK3CA and AKT1, which are well-known components of this pathway. Although these targets may not be novel in the broader context of cancer research, their specific modulation by rutin in TNBC offers new insights into potential therapeutic strategies.

Moreover, our study identified NF-κB as another key target of rutin in TNBC. The NF-κB signaling pathway is critical in regulating immune responses, cell proliferation, and apoptosis, all pivotal in cancer progression [21]. While NF-κB has been extensively studied, the interaction of rutin with this pathway in TNBC provides a novel angle for further exploration, especially considering the aggressive nature of TNBC and its resistance to conventional therapies. By focusing on these pathways, we highlight the potential of rutin as an adjuvant therapy, possibly enhancing the efficacy of existing treatments or reducing side effects.

The absence of viable therapy targets for TNBC is mainly attributed to the limited ability to diagnose the condition [22]. Therefore, it is crucial to understand the underlying mechanism of TNBC. The use of natural products is essential in exerting anti-tumor actions [23]. In this regard, Rutin’s pharmacological effects make it suitable for treating various types of cancer. Some studies have documented the chemopreventive properties of rutin against many cancers, including breast, colon, and colorectal cancers, as well as neuroblastoma, leukemia, hepatocellular carcinoma, and lung metastasis [24,25].

The network pharmacology approach combines systems biology with computer technology to provide a new direction for studying complex drug mechanisms. The development of network pharmacology allows researchers to shift from the traditional concept of “one drug, one target” to a model of “drug–target network” [26]. Therefore, the current study utilizes this method to study the potential molecular mechanism of rutin to treat TNBC. A total of 38 target genes related to TNBC were obtained by network pharmacological analysis. The PPI network, including IL6, TNF, INS, and EGFR, was used to screen four key targets.

IL-6 is a cytokine family member vital in producing specific cells, immunological responses, and inflammatory reactions. Prior research has demonstrated that IL-6 can stimulate the formation of blood vessels in tumors, facilitate the spread of tumor cells, and hinder the body’s natural defenses against tumors. As a result, IL-6 plays a role in initiating and advancing the growth of tumor cells. These tumor cells and tumor-associated macrophages can release high levels of IL-6 to form a vicious cycle [27].

TNF is secreted by mononuclear macrophages. It is recognized for displaying a diverse array of biological functions. A low concentration of TNF hinders the proliferation of some cancers, but a high concentration stimulates the production of IL-6 by stimulating monocytes, macrophages, lymphocytes, and neutrophils. This procedure elevates the concentration of IL-6 in the bloodstream, hence facilitating the progression of tumor growth [28]. IL-6 and TNF can affect all stages of tumor growth, such as initiation, promotion, progression, and metastasis [29].

INS is also a family member of the cell growth factor, which can enhance cellular proliferation capabilities and inhibit apoptosis. It is believed that there is a strong association between high levels of insulin and the occurrence and development of some tumors. All conditions of hyperinsulinemia, whether endogenous or exogenous, increase the risk of cancer [30]. Diseases that are characterized by hyperinsulinemia, i.e., obesity and diabetes, are further linked to an enhanced risk of endometrial, colorectal, and breast cancers [31,32]. In these cases, the possible mechanisms responsible for insulin-mediated tumor formation may include enhanced DNA synthesis leading to excessive cell growth, inhibition of apoptosis, and alteration of the sex hormone environment [33].

EGFR is a member of the transmembrane glycoprotein family. Its tyrosine kinase activity is widely recognized for regulating various molecular processes, including cell proliferation, differentiation, survival, and maintaining metabolic balance in the body. EGFR, upon binding to a ligand, can stimulate the activation of multiple cellular genes. This, in turn, leads to the promotion of cell proliferation, adhesion, metastasis, and vascular proliferation, all of which are intimately associated with the development of tumors [34]. EGFR has been involved in many types of cancers, like breast cancer, head and neck squamous cell carcinoma, lung, pancreatic, and colorectal [35,36,37,38,39]. About half of patients with TNBC and inflammatory breast cancer show overexpression of EGFR [40].

A similar study reported that rutin treatment led to a significant increase in Bax and caspase-3 levels, accompanied by decreased Bcl-2 expression, suggesting that rutin induces apoptosis in TNBC cells via the intrinsic mitochondrial pathway. These findings align with previous reports that flavonoids can trigger apoptosis through mitochondrial dysfunction [41]. Similarly, Liu et al. performed wound healing and transwell migration assays to evaluate the anti-metastatic potential of rutin in TNBC cells. Rutin significantly inhibited the migration and invasion of TNBC cells, which may be attributed to its downregulation of matrix metalloproteinases (MMPs) such as MMP-2 and MMP-9, proteins crucial for cancer metastasis [42].

The binding activity between the drug and the target was further verified by molecular docking. In *in vitro* experiments, it was found that rutin potentially inhibited the MDA-MB-231 cell growth in a dose and time-dependent way. The rate of apoptosis increased 48 h into the treatment. Rutin was shown to downregulate the mRNA and protein expression of four key targets in MDA-MB-231 cells by qPCR and Western blotting. Rutin may, therefore, actively inhibit TNBC by controlling EGFR, INS, TNF, and IL-6.

## 5 Conclusion

In summary, this study explored the potential molecular mechanism offered by rutin to inhibit breast cancer by using experimental authentication and network pharmacological prediction. The four primary targets are dispersed throughout many signaling pathways. As a result, rutin’s anti-cancer activity is due to a mixture of several targets and pathways. According to the findings of this investigation, more *in vivo* trials are needed. This study provides a theoretical foundation and research recommendations for the therapeutic application of rutin to treat TNBC.

## Abbreviations

BC: Betweenness centralities
BP: Biological process
CC: Cell component
CC: Closeness centralities
DC: Degree centralities
EC: Eigenvector centralities
ER: Estrogen receptor
GO: Gene ontology
HER2: Human epidermal growth factor receptor 2
KEGG: Kyoto Encyclopaedia of Genes and Genomes
LAC: Local average connectivity-based method
MF: Molecular function
NC: Network centralities
PPI: Protein–protein interaction
PR: Progesterone receptor
TNBC: Triple-negative breast cancer
